# Association between polymorphism rs6295 of *HTR1A* serotonin receptor gene and personality traits among athletes of combat sport

**DOI:** 10.5114/biolsport.2024.129478

**Published:** 2023-09-20

**Authors:** Kinga Humińska-Lisowska, Jolanta Chmielowiec, Krzysztof Chmielowiec, Aleksandra Strońska, Paweł Cięszczyk, Michał Spieszny, Jolanta Masiak, Milena Lachowicz, Olga Surała, Anna Grzywacz

**Affiliations:** 1 Faculty of Physical Education, Gdansk University of Physical Education and Sport, K.Górskiego St. 1, 80-336 Gdansk, Poland; 2 Department of Hygiene and Epidemiology, Collegium Medicum, University of Zielona Góra, 28 Zyty St., 65-046 Zielona Góra, Poland; 3 Independent Laboratory of Health Promotion, Pomeranian Medical University in Szczecin, 70-204 Szczecin, Poland; 4 Institute of Sports Sciences, the University of Physical Education in Krakow, 31-541 Kraków, Poland; 5 Neurophysiological Independent Unit, Department of Psychiatry, Medical University of Lublin, 1 Aleje Racławickie St., 20-059 Lublin, Poland; 6 Institute of Sport - National Research Institute, 01-982 Warsaw, Poland

**Keywords:** HTR1A, Genes, Athletes, Personality, rs6295, Sports predispositions

## Abstract

*HTR1A* (5-hydroxytryptamine receptor 1A) and its polymorphic variants are highly important for athletes in different aspects, allowing us to hypothesize their biological influences. Hence, to investigate at least part of the relationship mentioned in the case literature, it was decided to study the association of the selected *HTR1A* polymorphism with personality traits measured by the Temperament and Character Inventory (TCI). The participants consisted of 250 mixed martial arts (combat sport) athletes and 209 healthy male participants (control group). The personality traits were measured for the Revised Temperament and Character Inventory (TCI-R). Genetic material was isolated from whole blood collected from patients, and then all samples were genotyped using the real-time PCR method. Statistical analysis was performed using a 2 × 3 factorial ANOVA. The research revealed a statistically significant effect of a complex factor of rs6295 of the *HTR1A* serotonin receptor gene with combat sport/control and with Novelty Seeking (F_2,453_ = 6.126; *p* = 0.0024; η^2^ = 0.026) and Harm Avoidance (F_2,453_ = 3.709; *p* = 0.0252; η^2^ = 0.016). The presence of the *HTR1A* GG genotype (rs6295) was found to be associated with higher scores in self-management and lower scores in harm avoidance, indicating genetic predispositions in the strength group towards better results in combat sports.

## INTRODUCTION

The serotonin family includes at least 14 different 5-HT receptors [1]. The serotonin receptor 5-HTR1A (5-hydroxytryptamine receptor 1A) is a protein that regulates the release of serotonin by acting both as a presynaptic autoreceptor in the serotonergic neurons of the dorsal and medial raphe nuclei and as a postsynaptic heteroreceptor in non-serotonergic neurons. The 5-HTR1A receptor is encoded by a gene located on chromosome 5 (5q11.2–13) [2]. There is a common functional single nucleotide polymorphism (rs6295) in the promoter of the *HTR1A* gene, C (-1019) G. The G allele was associated with higher receptor expression, which led to increased negative feedback inhibition in the serotonergic neurons of the raphe nucleus (mediated by 5-HTR1A autoreceptors) and thus reduced serotonergic activity [3].

The 5-hydroxytryptamine 1A receptor gene (*HTR1A*) is located on chromosome 5 (5q12.3). In the central nervous system, the 5-HT1A receptor subtype is expressed in the cerebral cortex, hippo-campus, septum, amygdala and other limbic system structures in the raphe nucleus on the soma and dendrites of 5-HT neurons or postsynaptic receptors, basal ganglia and thalamus [4]. This gene is known for its functional rs6295 polymorphism, located in the promoter region, and regulates *HTR1A* transcription and region-specific modification of *HTR1A* expression [5]. In particular, the rs6295 G allele leads to higher expression of the *HTR1A* gene, leading to an increase in 5-HTR1A autoreceptors and a decrease in the level of the postsynaptic 5-HTR1A receptor [6]. The rs6295 polymorphism may therefore explain the risk of developing a mental illness. Haslacher et al. [7] noted that the C allele might protect against depressive mood in older endurance athletes (risk of development in the control group 30% vs. 2% of athletes carrying the C allele).

Given that athletes’ cognitive and psychological skills are critical factors in their ability to achieve a successful sports career, it is imperative to understand the involvement of serotonergic gene polymorphisms [8]. The serotonin 1A receptor is involved in memory and plays a crucial role in learning. It is found at high levels in the hippocampus and the raphe nucleus [4]. CC carriers also have better working and episodic memory [9].

The Temperament and Character Inventory (TCI) provides a comprehensive description of personality traits by measuring seven personality dimensions that are moderately heritable and associated with distinct brain networks and psychological characteristics [10]. The model measures four dimensions of temperament, which include basic emotional drives modulated by the hypothalamus and related limbic structures, and three dimensions of character, which rely on the self-regulation of emotions and attention to achieve the intended goals and values regulated mainly in the neocortex [10]. For example, high levels of self-targeting are associated with the executive attention system involving bipolar neurons in the anterior, frontal, and anterior cingulate [11]. Low Harm Avoidance is associated with diminished functional connectivity in the island’s projection network (i.e., the right anterior island with anterior cingulate gyrus and dorsolateral prefrontal cortex) [12]. Greater novelty seeking and less harm avoidance are associated with greater white matter volumes bilaterally in the cerebellum and cortex. Higher novelty scores are associated with larger caudate and bilateral pale nuclei volumes, while lower harm avoidance is related to reduced crust diffusivity as measured by diffusion tensor imaging [13].

*HTR1A* and its polymorphic variants are highly important for athletes in different aspects, allowing us to hypothesize their biological influences. Hence, to show at least part of the relationship mentioned in the case literature, it was decided to present the association of the selected *HTR1A* polymorphism with personality traits measured by the TCI test

## MATERIALS AND METHODS

### Research groups

Both athletes and controls were of Caucasian origin and living in one Polish region. The experiment was based on a group of 250 healthy Polish males (no prior history of substance dependency or psychosis) practising mixed martial arts (combat sports) aged 26.16 ± 8.34; mixed martial arts (MMA), n = 86; judo, n = 52; boxing, n = 52; karate, n = 26; kickboxing, n = 21; ju-jitsu, n = 13). Controls included 209 healthy (non-dependent and non-psychotic) Polish male volunteers aged 23.35 ± 5.35. All athletes and controls were white to reduce the possibility of racial bias and overcome any potential problems resulting from population stratification (Table 1). The study was supported by the National Science Centre of Poland (No. UMO-2017/27/B/NZ7/00204). The study was conducted according to the guidelines of the Declaration of Helsinki and approved by the Bioethics Committee for Clinical Research of the Regional Medical Society in Szczecin, Marii Skłodowskiej-Curie 11 Street (protocol no. 13/KB/VI/2016, 8 December 2016).

**TABLE 1:** Fundamental biological features of tested combat sports group and control group.

Feature	Combat sport n = 250 (M ± SD)	Control n = 209 (M ± SD)
Age (years)	26.16 ± 8.34	23.35 ± 5.35
Body mass (kg)	79.78 ± 13.76	81.85 ± 10.89
Height (cm)	178.34 ± 7.37	181.81 ± 6.22
BMI (kg/cm^2^)	24.62 ± 4.21	24.33 ± 4.30

*n* – number of subjects, M – mean, SD – standard deviation, BMI – Body Mass Index.

### DNA Isolation and Genotyping

A standard procedure of collecting venous blood was applied to obtain genomic DNA used for genotyping by the real-time PCR method. According to the standard manufacturer’s protocols, the genotyping of the rs6295 *HTR1A* gene was performed with fluorescence resonance energy transfer in the LightCycler 480 II System (Roche Diagnostic, Basel, Switzerland).

### Psychometric Tests

All participants in this study were asked to perform a psychometric test, precisely the Revised Temperament and Character Inventory [NO_PRINTED_FORM]. TCI-R is a self-report questionnaire assessing four temperaments (Harm Avoidance, Novelty Seeking, Reward Dependence, and Persistence) and three character higher-order dimensions (Self-Directedness, Cooperativeness, and Self-Transcendence) [14, 15]. Temperament refers to individual differences in percept-based habits and skills that are regulated by the limbic system [16, 17] and measured by four independently inherited dimensions that are moderately stable throughout life: Novelty Seeking (NS) refers to a tendency toward exploratory activities in response to novelty and is hypothesized to be mediated by a dopaminergic behavioural activation system; Harm Avoidance (HA) refers to pessimistic worrying in anticipation of problems that are hypothesized to be mediated by a serotonergic behavioural inhibition system; Reward Dependence (RD) is defined as a tendency to maintain behaviours in response to reward by others and is mediated by a noradrenergic behavioural maintenance subsystem; Persistence (PS) is an independent dimension and refers to a tendency to perseverance despite frustration and fatigue.

### Statistical Analysis

The distribution of rs6295 of the *HTR1A* serotonin receptor gene was tested by Hardy-Weinberg equilibrium (HWE) with the HWE software http://www.oege.org/software/hwe-mr-calc.html.

The variables that were analysed did not have a normal distribution. The results of the Mann-Whitney U test were used to determine the difference in the investigated features of Novelty Search, Harm Avoidance, Reward Dependence, Self-management, Cooperation abilities, and Self-transcendence skills.

Not all assumptions required for the ANOVA analysis were met. The speculation about the normal distribution was not fulfilled for all dependent variables, but the variance was the same (Levene’s test *p* > 0.05). Because the number of subjects in groups was also significant, it was decided to use multivariate analysis: 2 × 3 factorial ANOVA. The test was used to show an association between Novelty Seeking, Harm Avoidance, Reward Dependence, Self-management, Cooperation abilities, Self-transcendence skills results and the combat sport and control group, and the rs6295 of *HTR1A* polymorphism (personality traits × control and combat sports subjects × genetic feature).

The frequencies of genotypes and alleles of the rs6295 polymorphism of *HTR1A* in analysed groups were compared by the chi-square test. All analyses were performed using STATISTICA 13 (Tibco Software Inc, Palo Alto, CA, USA) for Windows (Microsoft Corporation, Redmond, WA, USA).

## RESULTS

The frequency distributions accorded with the HWE. There was no significant difference between combat sports subjects and control subjects (Table 2).

**TABLE 2:** Hardy-Weinberg equilibrium of *HTR1A* rs6295 polymorphism in combat sports subjects and controls group.

Group	*HTR1A* rs6295				

	Observed (Expected)	Alleles frequency	χ^2^	*p* value
combat sport, *n* = 250	GG	74 (80.1)	p allele freq (G) = 0.57 q allele freq (C) = 0.43	2.458	0.117

	GC	135 (122.8)			

	CC	41 (47.1)			

controls, *n* = 209	GG	45 (225.4)	p allele freq (G) = 0.49 q allele freq (C) = 0.51	1.413	0.234

	GC	113 (55.2)			

	CC	51 (3.4)			

*p* – statistical significance, χ^2^ – Chi^2^ test result, *n* – number of subjects.

Association analysis of genotypes and alleles of the *HTR1A* rs6295 polymorphisms and combat sport revealed statistically significant differences only in the co-dominant model in frequencies of genotypes (G/G 0.30 vs. 0.21; G/C 0.54 vs. 0.54; C/C 0.16 vs. 0.24; χ^2^ = 6.495; *p* = 0.039). In contrast, there was a statistically significant difference between the control group and combat sport group in the additive model (Cochran-Armitage trend test) in *HTR1A* rs6295 polymorphisms (Z = -2.539 *p* = 0.011). Significant differences in the frequency of *HTR1A* rs6295 gene alleles between combat sport subjects and the control group were found (G 0.57 vs. G 0.49, C 0.43 vs. C 0.51, χ^2^ = 5.900, *p* = 0.015) (Table 3).

**TABLE 3:** Frequency of genotypes and alleles of the rs6295 polymorphism of *HTR1A* in combat sports subjects and controls.

	Combat sport	Controls	Co-dominant model χ^2^ (*p* value)	OR (95% Confidence)	Additive model Cochran – Armitage trend test Z (*p* value)

*HTR1A* rs6295					
	*n* = 250 (%)	*n* = 209 (%)	6.495 (0.039)*	0.72 (0.46–1.14)	-2.539 (0.011)*

G/G	74 (30%)	45 (21%)			

G/C	135 (54%)	113 (54%)			

C/C	41 (16%)	51 (24%)			

G	283 (57%)	203 (49%)	5.900 (0.015)*	0.50 (0.29–0.87)*	

C	217 (43%)	215 (51%)			

*p* – statistical significance, χ^2^ – Chi^2^ test result, n – number of subjects,

* – significant statistical differences. OR – Odds Ratio

The means and standard deviations for Novelty Seeking, Harm Avoidance, Reward Dependence, Self-management, Cooperation abilities, and Self-transcendence skills in a group of combat sports subjects and control subjects are presented in Table 4. Compared to the controls, the case group subjects had significantly higher scores on Self-management (M 26.30 vs. M 24.01; *p* < 0.00001) and lower scores on the scales of Harm Avoidance (M 9.66 vs. M 11.60; *p* < 0.00001) (Table 4).

**TABLE 4:** Analysis of Novelty Seeking, Harm Avoidance, Reward Dependence, Self-Management, Ability to Cooperate, and Self-transcendence results in combat sports subjects and controls.

Temperament and Character Inventory (TCI)	Combat sport	Control	U Mann-Whitney test	*p* value

	(*n* = 250) M ± SD	(*n* = 209) M ± SD		
Novelty Seeking	20.03 ± 4.72	20.45 ± 4.36	-0.717	0.473
Harm Avoidance	9.66 ± 4.92	11.60 ± 4.57	-4.594	< 0.00001*
Reward dependence	10.03 ± 3.03	10.44 ± 2.95	-1.173	0.241
Self-management	26.30 ± 4.37	24.01 ± 5.03	4.850	< 0.00001*
Cooperative abilities	20.59 ± 4.59	19.83 ± 4.59	1.949	0.051
Self-transcendence skills	6.94 ± 3.59	7.09 ± 3.63	-0.419	0.675

M – mean, SD – standard deviation,

* – statistically significant between-group, n – number of subjects.

### Novelty Seeking and HTR1A rs6295

**FIG. 1:**
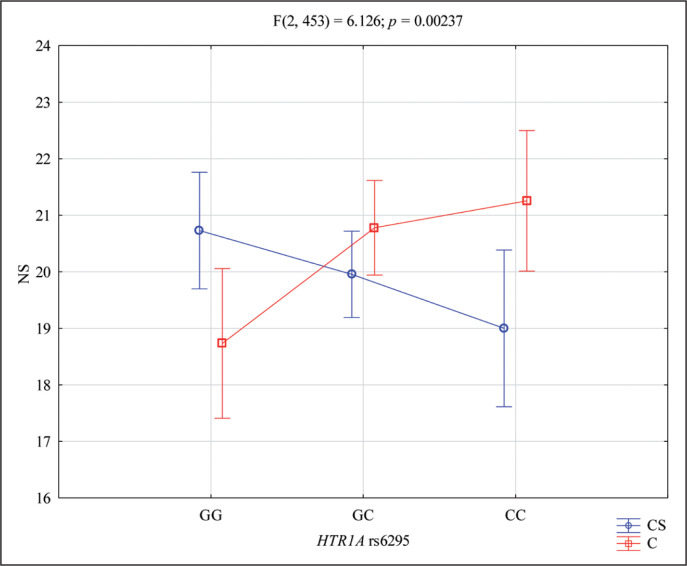
Interaction between CS (combat sport)/C (control) and rs6295 of *HTR1A* and NS – Novelty Seeking scale. NS – Novelty Seeking, *p* – statistical significance, CS – combat sport, C – control, GG and CC – genotypes (homozygotes), GC – genotype (heterozygote), G and C – alleles

The 2 × 3 factorial ANOVA results showed a statistically significant effect of the interaction Novelty Seeking and *HTR1A* rs6295 genotype of combat sport/control (F_2,453_ = 6.126; *p* = 0.0024; η^2^ = 0.026) (Table 5, Figure 1). Power calculation: the sample had more than 89% power to detect the combined factor of Novelty Seeking combat sport/control × *HTR1A* rs6295 and their interaction effect (about 2.6% of the phenotype variance). The results of the post hoc test are included in Table 6.

**TABLE 5:** The results of 2 × 3 factorial ANOVA for combat sports subjects and controls, incorporating: Novelty Seeking, Harm Avoidance, Reward dependence, Self-management, Ability to cooperate, Self-transcendence skills results and *HTR1A* rs6295.

Temperament and Character Inventory (TCI)	Group	*HTR1A rs6295*			ANOVA (interaction)	

		G/G (n = 119) M ± SD	G/C (n = 248) M ± SD	C/C (n = 92)M ± SD	Combat sport /Control × *HTR1A* rs6295; F (*p* value)	ɳ^2^
Novelty Seeking	combat sport; n = 250	20.73 ± 4.55	19.95 ± 5.09	19.00 ± 3.45	F_2,453_ = 6.126 (*p* = 0.0024)*	0.026

	Control; n = 209	18.73 ± 1.97	20.78 ± 5.00	21.25 ± 4.02		

Harm Avoidance	combat sport; n = 250	8.12 ± 4.26	10.20 ± 5.14	10.63 ± 4.78	F_2,453_ = 3.709 (*p* = 0.0252)*	0.016

	Control; n = 209	12.04 ± 4.30	11.53 ± 4.84	11.37 ± 4.22		

Reward dependence	combat sport; n = 250	9.99 ± 2.97	9.98 ± 3.03	10.29 ± 3.18	F_2,453_ = 0.130 (*p* = 0.8777)	0.0006

	Control; n = 209	10.22 ± 3.01	10.31 ± 2.97	10.94 ± 2.87		

Self-management	combat sport; n = 250	25.88 ± 4.20	26.51 ± 4.49	26.39 ± 4.33	F_2,453_ = 0.880 (p = 0.4154)	0.004

	Control; n = 209	24.60 ± 4.55	23.86 ± 5.01	23.82 ± 5.51		

Cooperative abilities	combat sport; n = 250	20.12 ± 4.57	20.60 ± 4.69	21.41 ± 4.25	F_2,453_ = 0.115 (*p* = 0.8913)	0.0005

	Control; n = 209	19.62 ± 4.27	19.70 ± 4.85	20.33 ± 4.28		

Self-transcendence skills	combat sport; n = 250	6.40 ± 3.55	7.21 ± 3.62	7.02 ± 3.33	F_2,453_ = 1.233 (*p* = 0.2923)	0.005

	Control; n = 209	6.55 ± 3.48	6.87 ± 3.80	8.05 ± 3.26		

M – mean, SD – standard deviation, n – number of subjects, *p* – statistical significance,

* – significant statistical differences, ɳ^2^ – eta square

**TABLE 6:** Post hoc LSD (least significant difference) test of interactions between combat sport/controls, *HTR1A* rs6295, and the Novelty Seeking, Harm Avoidance scale.

***HTR1A* rs6295 and Novelty Seeking scale**						

	{1} 20.73	{2} 19.96	{3} 19.00	{4} 18.73	{5} 20.78	{6} 21.26

Combat sport *HTR1A* G/G {1}		0.2366	0.0498*	0.0198*	0.9422	0.5232

Combat sport *HTR1A* G/C {2}			0.2360	0.1166	0.1535	0.0807

Combat sport *HTR1A* C/C {3}				0.7846	0.0313*	0.0177*

Control *HTR1A* G/G {4}					0.0105*	0.0066*

Control *HTR1A* G/C {5}						0.5323

Control *HTR1A* C/C {6}					

***HTR1A* rs6295 and Harm Avoidance scale**						

	{1} 8.12	{2} 10.21	{3} 10.63	{4} 12.04	{5} 11.53	{6} 11.37

Combat sport *HTR1A* G/G {1}		0.0024*	0.0065*	0.00001*	0.000002*	0.0002*

Combat sport *HTR1A* G/C {2}			0.6125	0.0243*	0.0284*	0.1339

Combat sport *HTR1A* C/C {3}				0.1672	0.2980	0.4563

Control *HTR1A* G/G {4}					0.5376	0.4869

Control *HTR1A* G/C {5}						0.8424

Control *HTR1A* C/C {6}						

* – significant statistical differences. For these variables, G/G and C/C – genotypes (homozygotes), G/C – genotype (heterozygote), G and C – alleles, 1 – G/G, 2 – C/C, 3 – C/C, 4 – G/G, 5 – G/C, 6 – C/C, *HTR1A* – 5-hydroxytryptamine receptor 1A

### Harm Avoidance and HTR1A rs6295

**FIG. 2:**
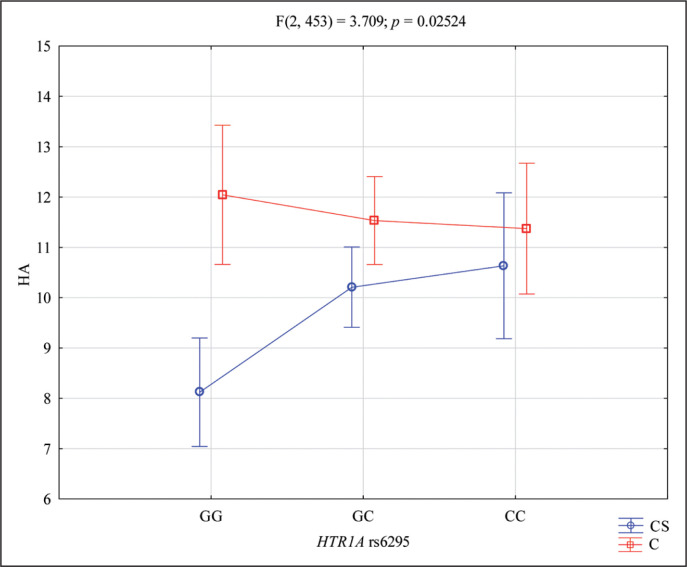
Interaction between CS (combat sport)/C (control) and *HTR1A* rs6295 and HA – Harm Avoidance scale. HA – Harm Avoidance, *p* – statistical significance, CS – combat sport, C – control, GG and CC – genotypes (homozygotes), GC – genotype (heterozygote), G and C – alleles

The results of 2 × 3 factorial ANOVA showed a statistically significant effect of the interaction Harm Avoidance and *HTR1A* rs6295 genotype of combat sport/control (F_2,453_ = 3.709; *p* = 0.0252; η^2^ = 0.016) (Table 5, Figure 2). Power calculation: the sample had more than 68% power to detect the combined factor of Harm Avoidance combat sport/control × *HTR1A* rs6295 and their interaction effect (about 1.6% of the phenotype variance). The results of the post hoc test are included in Table 6.

## DISCUSSION

The present study focused on gene variants that potentially influence the modulation of emotion-regulating systems, particularly the gene responsible for encoding components of the serotonergic system.

Genetic factors are widely recognized to influence various phenotypic traits related to sports performance and the achievement of elite athletic status [18]. More than 200 genetic variants are associated with physical performance, and 155 are linked to elite athlete status [19]. Undoubtedly, psychological factors play a significant role, especially at the sport’s highest level [20]. Between 70% and 85% of successful and unsuccessful athletes can be distinguished using the general psychological personality structure and mood state [21]. For example, the authors recently noted an association between the *DAT1* VNTR (Variable Number of Tandem Repeats) variant and lower anxiety levels in a group of tested athletes [22]. Also, it was observed that temperamental characteristics may indicate higher resilience of the nervous system in combat athletes in comparison to non-athletes [23]. However, the genetic foundation of the emotional and mental features predisposing to outstanding athletic performance is still elusive.

It was observed previously that mentally tough athletes effectively use motor skills in stressful situations to perform cognitive tasks better [24]. It is widely assumed that mental qualities such as planning skills, persistence, patience, mental strength, ambition and pursuit of leadership, stress-coping, preventing anxiety-like behaviour, and avoiding impulsivity and uncontrolled aggressiveness facilitate athletic training and success in competition [25]. Competition, especially its crucial moments, requires maximum physical and mental mobilization from the athlete. Training must comprise physical and psychological preparation to achieve a high level of sports efficiency. Since physical differences among toplevel athletes are often barely noticeable, their ability to deliver optimal physical and mental performance precisely when required could be the decisive factor determining their actual standing [26].

Competition-facilitating behaviour and the low-stress response described above differentiate athletes from sedentary subjects [27]. Peplonska et al. [28], to search for the putative genetic background of mental characteristics, analysed 67 polymorphic variants in 28 genes potentially associated with behaviour, mood, and emotion expression plausibly related to competitiveness. These variants have previously been considered mainly in addiction, depression, suicidal tendencies, the aetiology of numerous psychosomatic and psychiatric disorders, and aggressive behaviour.

Both variants have been analysed previously, especially in the behavioural context. *HTR1B* rs11568817 has been associated with alcohol and drug abuse, whereas *HTR2C* rs3813929 has been associated with increased resistance to obesity and type II diabetes, but also migraine, schizophrenia, and depression [29]. Genetic association studies cannot point to particular molecular mechanisms underlying various phenotypes, as mentioned above, especially given the pleiotropic nature of genes. Mechanisms linking genetic variants with sport-related phenotypes, taking into account the function of genes bearing the variants, can only be hypothesized. It could be speculated that the common elements in such different behaviours as, e.g., addiction and sports competition, are based on a craving for excitement. However, explaining genotype-phenotype relationships requires in-depth functional studies [28].

In several studies of twins, the relative share of genetic factors in personality traits ranged from 40 to 60% [30]. In the biosocial theory of personality, Cloninger, using the Temperament and Character Inventory (TCI), found that temperament dimensions are especially related to activity in certain central neurotransmitter systems. However, the dimension of the character of self-transcendence is the most stable in the time dimension of TCI and the dimensions of TCI, showing the greatest variability among individuals [31]. TCI self-transcendence consists of three subscales: Spiritual Acceptance, Transpersonal Identification, and Self-Forgetting.

The polymorphism of the serotonin transporter (5-*HTTLPR*) promoter, consisting of a variable number of tandem repeats (VNTR), has been extensively studied in relation to personality disorders and mental disorders. Functional studies have shown that 5-*HTT* gene transcription is differently modulated by long and short 5-*HTTLPR* variants, where the short variant is associated with lower 5-*HTT* expression and lower 5-HT reuptake activity [32].

In this study, we report that the character of the self-transcendence personality, estimated using the Cloninger TCI, was significantly related to the genotype of the 5-*HTTLPR* gene polymorphism, as reported by Heils et al. [33], as well as to the variable repeat [CAAA] in the second intron of the AP-2β transcription factor gene described by Moser et al. [34]. The associations, however, occurred only in boys. Data supporting the results presented by Borg et al. [35] showed that 5-HT1A (11C) WAY100635 receptor-ligand binding was associated with the expression of Self-transcendence, but only in boys. The Self-transcendence dimension includes several aspects of religious behaviour, subjective experience, and individual worldview. In the Minnesota Study of Twins Reared Apart, religious heredity was found to be approximately 40% [36][34]. Links to multiple monoaminergic gene alleles further support genetic solid regulation of the character trait Self-transcendence (spiritual acceptance) with 5-HT1A receptor [34], 5-HT2A and 5-HT6 [37], and dopamine D4 polymorphisms.

Undoubtedly, psychological factors play a significant role, especially at the sport’s highest level. Additionally, many reports emphasize the positive effect of exercise on the anti-depressant state [38]. Between 70% and 85% of successful and unsuccessful athletes can be distinguished using general psychological measures of personality structure and mood state [21]. However, the genetic foundation of the emotional and mental features predisposing to outstanding athletic performance remains elusive.

Several studies have investigated a potential association between the C(−1019)G polymorphism and various personality traits; using the revised five-factor Personality Inventory (NEO-PI-R) and the Tridimensional Personality Questionnaire (TPQ), higher scores for Neuroticism and Harm Avoidance were found in carriers of the G allele compared with C allele carriers [39], while other studies did not find any significant association between neuroticism and this SNP [40].

In a study of a Hungarian population, G/G carriers displayed significantly higher impulsivity levels than G/C and C/C carriers [40].

Furthermore, the C(-1019)G polymorphism was investigated in relation to personality traits. The G allele was associated with different anxiety- and depression-related personality traits in a nonclinical German population, such as neuroticism and harm avoidance [39]. However, such an association was not always supported. Another study also failed to report an association between the C(-1019)G polymorphism and different personality traits in a German population of suicide attempters and healthy controls and an Italian population of patients diagnosed with a mood disorder [16].

This study revealed that personality traits are an area of note in analysing genetics in a dispute. As it was observed in this analysis, the results of 2 × 3 factorial ANOVA showed a statistically significant effect of the interaction between Novelty Seeking and *HTR1A* rs6295 genotype of combat sport/control subjects. This relationship is clearly illustrated in Figure 1. However, another feature also deserves attention – Harm Avoidance – the results of 2 × 3 factorial ANOVA showed a statistically significant effect of the interaction between Harm Avoidance and *HTR1A* rs6295 genotype of combat sport/control subjects. There was more than 68% power to detect the combined factor of Harm Avoidance combat sport/control × *HTR1A* rs6295 and their interaction effect.

Genetic polymorphism may be used as an additional scientific tool to assist athletes and coaches in sport selection [17].

## CONCLUSIONS

The study reveals the validity of analysing connections between personality traits and selected gene polymorphisms in athletes, a relatively new field. The presence of the *HTR1A* GG genotype (rs6295) is associated with higher self-management scores and lower harm avoidance scores, indicating genetic predispositions in the strength group for better results in combat sports. Despite limitations such as a small sample size and limited analysis of polymorphic variants, the findings already demonstrate significant associations. Further research with larger participant groups and expanded gene analysis is needed to explore these relationships more comprehensively.
